# Reducing the Cost of Being the Boss: Authentic Leadership Suppresses the Effect of Role Stereotype Conflict on Antisocial Behaviors in Leaders and Entrepreneurs

**DOI:** 10.3389/fpsyg.2021.760258

**Published:** 2021-11-16

**Authors:** Lucas Monzani, Guillermo Mateu, Alina S. Hernandez Bark, José Martínez Villavicencio

**Affiliations:** ^1^Ivey Business School, University of Western Ontario, London, ON, Canada; ^2^Department of Finance, Law and Control, Burgundy School of Business, University Bourgogne Franche-Comté, CEREN, EA 7477, Dijon, France; ^3^Department of Accounting, University of Valencia, Valencia, Spain; ^4^Department of Social Psychology, Goethe University Frankfurt, Frankfurt, Germany; ^5^Instituto Tecnológico de Costa Rica, Cartago, Costa Rica

**Keywords:** entrepreneur role stereotype, female entrepreneurship, gender-entrepreneur role incongruence, leader-entrepreneur role incongruence, antisocial behaviors, economic games

## Abstract

What drives entrepreneurs to engage in antisocial economic behaviors? Without dismissing entrepreneurs’ agency in their decision-making processes, our study aims to answer this question by proposing that antisocial economic behaviors are a dysfunctional coping mechanism to reduce the psychological tension that entrepreneurs face in their day-to-day activities. Further, given the overlap between the male gender role stereotype and both leader and entrepreneur role stereotypes, this psychological tension should be stronger in female entrepreneurs (or any person who identifies with the female gender role). We argue that besides the well-established female gender role – leader role incongruence, female entrepreneurs also suffer a female gender role – entrepreneur role incongruence. Thus, we predicted that men (or those identifying with the male gender role) or entrepreneurs (regardless of their gender identity) that embrace these roles stereotypes to an extreme, are more likely to engage in antisocial economic behaviors. In this context, the term antisocial economic behaviors refers to cheating or trying to harm competitors’ businesses. Finally, we predicted that embracing an authentic leadership style might mitigate this effect. We tested our predictions in two laboratory studies (Phase 1 and 2). For Phase 1 we recruited a sample of French Business school students (*N* = 82). For Phase 2 we recruited a sample of Costa Rican male and female entrepreneurs, using male and female managers as reference groups (*N* = 64). Our results show that authentic leadership reduced the likelihood of entrepreneurs and men of engaging in antisocial economic behaviors such as trying to harm one’s competition or seeking an unfair advantage.

## Introduction

For a time, Elizabeth Holmes was a true inspiration for female entrepreneurs. Young, charismatic, and successful in Silicon Valley, the “girl boss” reigned triumphant over a sector infamous for its hyper-masculine “bro culture” (Cook, 2020). Yet, as CEO of Theranos, Mrs. Holmes faked the results of clinical trials and reported doctored information to her shareholders. The actions of Holmes and other unethical female leaders created a headache for those scholars that related the female anatomical sex to a higher frequency of ethical behaviors at work (Borkowski and Ugras, 1998; Whitley et al., 1999; Childs, 2012).

As Mrs. Holmes and many other young entrepreneurs in the health sector found out the hard way (e.g., Mr. Martin Shkreli – “the Pharma Bro”), unethical business practices do not pay in the long run. While antisocial economic behaviors might bring results in the short term, engaging in antisocial economic behaviors leads to adverse long-term outcomes for leaders and entrepreneurs, their employees, their ventures capitalists, and other stakeholders. Without dismissing a person’s agency as a driver of unethical behavior in leaders and entrepreneurs, we asked ourselves if Mrs. Holmes’ unethical behavior was just a matter of individual differences (e.g., anatomical sex)? Or could these antisocial economic behaviors be a dysfunctional way of copying with the “cost of being the boss”?

There are three reasons why answering our research questions matters. First, such understanding would explain recurring issues in the entrepreneurship literature (Hughes et al., 2012; Jennings and Brush, 2013), such as why men are more likely to become entrepreneurs than women. Second, it would explain why some entrepreneurs decide to engage in unethical business practices, such as the antisocial economic behaviors that Mrs. Holmes and Mr. Shkreli displayed while leading their ventures. Third, scholars might use our findings to design interventions that deter entrepreneurs from engaging in unethical business practices and prevent future harm to shareholders and other stakeholders.

Role Congruency Theory (RCT; Eagly and Karau, 2002) is a valuable theoretical anchor for our research efforts. RCT explains well why women and other minorities suffer a double bind and prejudice when seeking or occupying leadership roles. Unfortunately, RCT does not explain the nuances of how this mechanism would work outside the traditional context of corporate firms. Whereas RCT would explain why women might suffer from reduced access to venture capital, it does not explain why female leaders might engage in the hyper-masculine antisocial economic behaviors that Mrs. Holmes displayed as the founder of her firm. Thus, by extending RCT to the female entrepreneurship arena, we provide a valuable theoretical contribution that informs the practice of leadership and entrepreneurship.

The main objective of this study is to determine if female entrepreneurs make antisocial decisions as a dysfunctional way of copying with the psychological tension created by simultaneously occupying incongruent social roles. To this end, we conducted two laboratory studies in two western countries. In a sample of business school students, Phase 1 tests our predictions about the effects of role conflict among three future roles on antisocial decisions employing two behavioral games. Phase 2 tests main and interactive effects of the same roles on antisocial decisions in a sample of Costa Rican entrepreneurs and managers.

## Literature Review

A myriad of studies supported the propositions of RCT (Eagly and Karau, 2002). RCT proposes that women suffer a prejudice that prevents them from (a) reaching leadership roles in corporations, and by which (b) women are evaluated more harshly than men in a leadership position (Koenig et al., 2011). RCT invokes cognitive dissonance as the psychological mechanism driving said prejudice toward female leaders. RCT claims that when the characteristics of a person occupying a role misalign with the stereotypical expectations toward a given role, “this inconsistency lowers the evaluation of the group member as an actual or potential occupant of the role” (Eagly and Karau, 2002, p. 574).

Following the logic behind RCT, we argue that female entrepreneurs suffer a similar (or even stronger) prejudice than female managers, given that “entrepreneur” is also a social role (“think entrepreneur – think male;” Laguía et al., 2018). We expect entrepreneurs to suffer the effects of an additional cognitive dissonance (regardless of their anatomical sex or gender identity), which arises from the conflicting stereotypical expectations toward the leader and entrepreneur role. This logic also suggests that female entrepreneurs will suffer conflicting expectations toward three instead of two social roles.

**FIGURE 1:**
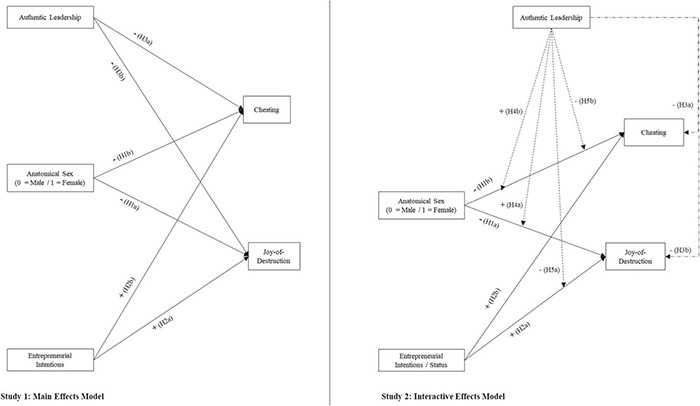
Theoretical model describing main (left) and interactive effects (right).

This “triple bind and prejudice” should then result in a stronger psychological tension than the one suffered by their male counterparts. Whereas there are always functional ways of reducing psychological tension, antisocial economic behaviors seem to result from dysfunctional copying mechanisms (self-stereotyping; in-extremis identity trade-off). We unpack this last claim in the following section and summarize our predictions in Figure 1.

### Embracing Role Stereotypes In-Extremis and Antisocial Economic Behaviors

Role stereotypes describe the “ideal” representations of social roles in a social group or culture. In turn, these ideal representations are incorporated into a person’s sense of self through a psycho-social process called socialization (Hartley, 1959). Once a role stereotype is internalized into the self, it drifts from the stream of consciousness and starts eliciting automatic behavioral as responses to external stimuli. Because role stereotypes only describe “ideal” representations, said representations are susceptible to change across time (history) and space (cultures), and might always reflect the reality behind the stereotype. The present study focuses on three Western and contemporary role stereotypes and predicts what occurs if these stereotypes are embraced in-extremis.

For a virtue ethics view, the “in-extremis” adjective refers to virtues becoming vices due to an excessive display of said virtue. For example, an excessive display of the three character strengths that compose the virtue of Courage (Bravery, Persistence, and Integrity) might lead to recklessness, zealously, and self-righteousness (Crossan et al., 2013, 2017). Virtue would reside using one’s practical wisdom to avoid any “in-extremis” behavior. Embracing in-extremis a social role is consistent with what behavioral economics defined as *self-stereotyping* (Latrofa et al., 2010). For behavioral economists, self-stereotyping occurs when an “agent perceives himself or herself as an interchangeable exemplar of a social group rather than as a unique individual” (Hernandez-Arenaz, 2020, p. 2). Social psychologists and behavioral economists seem to agree that internalized stereotypes affect an agent’s economic behavior.

There are psychological risks associated with self-stereotyping. For example, self-stereotyping into a leader stereotype and embracing in-extremis its hyper-assertive prescriptions might result in seeking unethical ways to fulfill organizational goals (Ordóñez et al., 2009). Similarly, self-stereotyping into an entrepreneur role and embracing in-extremis its hyper-competitive prescriptions might result in lying to secure additional venture funding. Finally, self-stereotyping into the male gender role and embracing in-extremis the dominant and assertive behavioral prescriptions can elicit “toxic masculinity” behaviors (e.g., misogyny, homophobia, violence; Harrington, 2020).

#### The Female Gender Role Stereotype

In Western societies, the female role describes nurturing characteristics, such as gentleness, empathy, and support. Instead, the male gender role stereotype describes agentic characteristics, such as results-orientation and concern for advancing one’s social status. The female gender role stereotype prescribes communal behaviors (e.g., concern about the well-being of others). Instead, the male gender role stereotype describes agentic behaviors (e.g., being assertive and dominant; Abele et al., 2008; Hernandez Bark et al., 2014, 2015; March et al., 2016; Hentschel et al., 2019). As mentioned above, any behavior which deviates from these stereotypical role expectations will likely elicit some form of social backlash, and particularly for women leading in organizational contexts (Gloor et al., 2018).

Adherence to gender role stereotypes can also be identified in economic games. For example, women demonstrated greater aversion toward lying for a small monetary benefit (Childs, 2012) and lower dishonesty levels than men (Friesen and Gangadharan, 2012). However, Ezquerra et al. (2018) found no gender differences in a cheating game. It follows that men (or those who identify with the male role) who self-stereotype and embrace their gender role in-extremis will more likely try to assert their dominance at work. Such a need for dominance will likely elicit antisocial economic behaviors, such as cheating and trying to harm their competition, that is, displaying *toxic masculinity*.

*Hypothesis 1a*: Men (or those who identify with the male gender) will be more likely (a) to display antisocial behaviors aimed at harming their competition than women (or those who identify with the female gender).

*Hypothesis 1b*: Men (or those who identify with the male gender role) will be more likely (a) to seek an unfair advantage by cheating than women (or those who identify with the female gender role).

#### The Entrepreneur Role Stereotype

The entrepreneur role stereotype collects competition-oriented traits that are seen as predictors of entrepreneurial success (need for achievement, generalized self-efficacy, individualism, risk-taking, proactive personality; Rauch and Frese, 2007; Frese and Gielnik, 2014). Further, the entrepreneur stereotype prescribes the pursuit of wealth through the creation of new transactions (Smith et al., 2009).

Whereas competition is an inherent part of doing business, fair competition does not require entrepreneurs and business leaders to engage in antisocial economic behaviors. Yet, we claim that embracing the entrepreneur role stereotype in-extremis should elicit behaviors would appear “rational” in the traditional economic sense of the world, such as maximizing individual profits whenever possible, free-riding, and not contributing to social causes, unless when it brings an advantage for entrepreneurs. Further, another consequence of embracing in extremis the entrepreneurial role would be a higher likelihood of misrepresenting information, for example, to increase the chances to “win” further venture funding.

*Hypothesis 2a*: Entrepreneurs (or aspiring entrepreneurs) will be more likely (a) to display antisocial behaviors aimed at harming their competition than managers (or aspiring managers).

*Hypothesis 2b*: Entrepreneurs (or aspiring entrepreneurs) will be more likely to seek an unfair advantage by cheating than managers (or aspiring managers).

#### The Leader Role Stereotype

The leader role stereotype collects the implicit beliefs of a given social group about the “ideal” attributes that describe successful leaders. Transactional leadership is a mainstream leadership style that collects such a pattern of behaviors in western countries. Some transactional behaviors include preserving the *status quo* by rewarding with justice and actively reducing deviations from existing norms and procedures (Bass, 1985).

This transactional, behavioral pattern underlies the classic view of rational management (Zehnder et al., 2017). Again, we argue that self-stereotyping and embracing the leader role stereotype in-extremis would result in agentic behaviors aimed at increasing efficiency *at any cost* (even through unethical business practices). Stated differently, the preference for antisocial economic behaviors in business managers would evidence an in-extremis embracing of the leader role stereotype.

The above stereotypical expectations toward the leader role remain deeply rooted in Western Societies. However, the corporate scandals that led to the 2008 financial crisis challenged the perceived value of pursuing profit *at and cost* in favor of a more sustainable approach to doing business. Today, scholars care as much for “what” constitutes effective leadership as much as the “how” leaders deliver performance (Gandz et al., 2010; Monzani et al., 2016, 2019, 2021b). Authentic leadership emerged as one of many positive alternatives to the prevailing western stereotypical view of leadership (Monzani and Van Dick, 2020).

Authentic Leadership (AL) should be of interest to entrepreneurs as well. Entrepreneurs who display authentic leadership behaviors tend to feel more self-expressive when leading their ventures (Jensen and Luthans, 2006b). Further, authentic entrepreneurs elicit employee affective commitment, satisfaction, and citizenship behaviors (Jensen and Luthans, 2006a). Despite these early studies, the study of authentic entrepreneurship is in its infancy (Lewis, 2013).

One dimension of the authentic leadership style is particularly relevant for our study of antisocial economic behaviors. The dimension of “internalized moral perspective” (IMP) majorly prescribes agentic behaviors by upholding moral behaviors independently of contextual pressures (e.g., “making difficult decisions based on high standards of ethical conduct”).

The ethical aspect of the IMP resonates well this some of the agentic prescriptions of the male gender role (such as being assertive). Yet, an IMP reminds leaders about the importance of adhering to existing social norms despite contextual pressures to act unethically (Monzani et al., 2015). Prior studies have shown that the more frequently leaders act coherently with their internalized moral perspective, the less likely they will engage in antisocial behaviors. Further, at least theoretically, the other three communal dimensions of AL would not prescribe antisocial behaviors. Thus, we can extend that logic into our hypotheses to claim that adopting an authentic leadership style tends to des-incentivize the display of hyper-competitive antisocial economic behaviors in favor of moral action (Hannah et al., 2011, 2014).

*Hypothesis 3a*: As the frequency of authentic leadership behaviors increases, the likelihood of displaying antisocial economic behaviors aimed at harming their competition will decrease.

*Hypothesis 3b*: As the frequency of authentic leadership behaviors increases, the likelihood of displaying antisocial economic behaviors seeking unfair advantage through cheating will decrease.

### Mitigating the Effect of Stereotypical Role Expectations on Antisocial Economic Behaviors

Due to our proposed “triple bind and prejudice,” female entrepreneurs should suffer a stronger prejudice than male entrepreneurs. Moreover, reconciling the stereotypical expectations toward three social roles should result in more psychological tension than their female manager counterparts. The unfortunate stereotype “Think entrepreneur – think male” (Laguía et al., 2018) captures this additional source of psychological tension. Female entrepreneurs usually struggle with limited access to venture capital, increased work-family conflict, and lack of spousal support (Das, 2000) unless they start ventures in areas congruent with stereotypical gender role expectations (e.g., social entrepreneurship; Carter et al., 2015). Increased psychological tension due to role expectations would explain why many female business students prefer a managerial position in the corporate world than starting a new venture (Jennings and Brush, 2013).

Another source of tension is the need to reconcile others’ conflicting expectations of how entrepreneurs and managers should act (regardless of one’s gender identity). For example, venture capitalists tend to expect entrepreneurs to be innovative by “moving fast and breaking things.” Yet, the same venture capitalists expect said entrepreneurs to be efficient by “moving slow and organizing things” (Taneja, 2019). “Organizing things” refers to developing a business strategy, making calculated decisions, and shaping norms that reduce the uncertainty inherent to any venture. Thus, to validate entrepreneurs as leaders, stakeholders demand from entrepreneurs to be visionary and managerial *at the same time* (Rowe, 2001).

To reduce the psychological tension resulting from these conflicting role expectations, individuals usually engage in “identity trade-offs” (Knapp et al., 2013). A role identity trade-off refers to following the stereotypical behavioral prescriptions of a given role (e.g., entrepreneur) to reduce the social pressure to conform to an opposing social role (e.g., gender, leader). For example, many women see in starting a new business (entrepreneur role) a functional alternative to “break free” from the societal forces that hinder their access to executive roles within corporations (leader role; Ryan and Haslam, 2007; Cook and Glass, 2014). In this way, female entrepreneurs can reduce the pressure of prevailing gender role stereotypes (female gender role), by having more latitude to balance entrepreneurial activities with their family life activities.

### Authentic Leadership, Gender, and Entrepreneurial Status

From a gendered view of leadership, the authentic leadership style prescribes both agentic and communal behaviors, and thus can be classified as an androgynous style (Monzani et al., 2015). Three out of four authentic leadership dimensions to some extent overlap with the Transformational Leadership style (Banks et al., 2016), and thus prescribe communal leader behaviors (Self-awareness, Balanced Processing of Information, and Relational Transparency). More precisely, Self-awareness refers to the awareness of goals, emotions, and needs of both self *and others*. Balanced Processing of Information refers to considering *different viewpoints* before making decisions. Finally, Relational Transparency refers to establishing clear and transparent relations *with others* (Walumbwa et al., 2008).

The communal dimensions of the authentic leadership style align well with the female gender role stereotype. Such alignment could explain the findings of a recent meta-analysis, suggesting a conceptual and empirical overlap between authentic and transformational leadership when predicting several positive, growth-oriented followers outcomes (Banks et al., 2016). The overlap between transformational leadership and authentic suggests that some of the insights of RCT might as well apply to the communal dimensions of AL, and thus allows predicting potential interactive effects between gender and leader role stereotypes.

The fact that such overlap exists might have implications for entrepreneurs as well. For example, as entrepreneurs increase the frequency of their authentic leadership behaviors when running their ventures, in turn, should increase entrepreneurs’ concern on how their actions impact others. Such concern should reduce the likelihood of displaying antisocial behaviors. Therefore, in this follow-up study, we propose the two additional hypotheses. The right panel of panel of Figure 1 summarizes our additional predictions:

*Hypothesis 4*: Authentic leadership moderates the effect of the male gender role on the likelihood of harming others’ firms (H4a) and cheating (H4b). As the frequency of authentic leadership behaviors increase, men will be less likely to display said antisocial behaviors.

*Hypothesis 5*: Authentic leadership moderates the effect of the entrepreneurial role on the likelihood of harming others’ firms (H5a) and cheating (H5b). As the frequency of authentic leadership behaviors increase, men will be less likely to display said antisocial behaviors.

## Methods

We tested our hypotheses in two laboratory studies. Our first laboratory study (Phase 1) was conducted in a sample of French Business school students (*N* = 82). However, this sample had some limitations (culturally heterogeneous, aspiring leaders and entrepreneurs). To address such limitations, we conducted a follow-up study (Phase 2). During Phase 2, we re-tested our predictions in a more homogeneous sample and explored interactive effects among predictors. More precisely, we needed a societal context that valued “tradition” (i.e., reinforces the female gender role stereotype) and “benevolence” (i.e., preserving and enhancing the welfare of those with whom one is in frequent contact).

Our rationale for choosing such a societal context is that we anticipate that in societies that simultaneously embrace the universal values of “tradition” and “benevolence,” the psychological tensions between conflicting role stereotypes would become more salient for female entrepreneurs than in other societies. On one side, a traditional society tends to pressure female citizens to find meaning by starting a family rather than a business.

On the other side, benevolent societies tend to value ventures that transcend the pure and single pursuit of profit. We would not expect the same level of psychological conflict in societies that score high in the universal value of benevolence and self-direction. Benevolence and self-direction do not seem to be at odds (i.e., should not create such a strong psychological tension when women occupy an entrepreneurial role).

In prior studies, Costa Rica scored 30.4% higher than Canada in the universal value of “Tradition” (*M* = 5.25, *SD* = 1.47 vs. *M* = 4.57, *SD* = 1.23 respectively). However, in the same study Costa Rica also matched the US in the value of “Benevolence” (*M* = 6.20, *SD* = 1.05 vs. *M* = 6.19, *SD* = 0.96 respectively; Schultz and Zelenzy, 1999). With such findings in mind, the Costa Rican society would be sending ambiguous signals about the value of entrepreneurship to their female citizens (or those who identify with the female gender role).

As a result of such mixed signals and ambiguity, Costa Rica seems to be a pristine context to explore how female entrepreneurs reconcile the conflicting pressure of multiple social stereotypes. Further, it allows us to test if female entrepreneurs will display antisocial economic behaviors when leading their ventures in a society that does not value nor socially reward such antisocial economic behaviors. On these grounds, we chose to conduct the second laboratory study (Phase 2) in Costa Rica. The second laboratory study is based on a sample of Costa Rican male and female entrepreneurs, taking male and female managers as reference groups (*N* = 64).

### Sample

For phase 1, our sample consisted of 82 students who attended business management courses at a French School of Business. The mean age was 22.37 years (*SD* = 1.95). A large part of our sample consisted of international students (57.3%). Thirty-five participants (42.7%) came from Mediterranean countries, Thirty (36.6%) from Asian countries, nine (11.0%) from Latin-American countries, three (3.7%) came from African nations, and three (3.7%) from Middle Eastern countries, two participants did not indicate their nationality. Twenty-one participants were male, sixty female, and one participant did not indicate his or her anatomical sex. After removing cases with missing data, our final sample for phase 1 consisted of 77 participants.

To address the limitations of Phase 1, in Phase 2 we invited traditional entrepreneurs (*N* = 20; 55.0% female) and managers from a public organization (*N* = 44; 43.2% female) to participate in our laboratory study. Entrepreneurs’ age *M* = 40.11; *SD* = 10.42 and Managers’ age was *M* = 44.58, *SD* = 8.28. Both the entrepreneurs (*M* = 8.44, *SD* = 10.54) and managers (*M* = 18.70, *SD* = 19.13) had several employees under their charge. 65.0% of our entrepreneurs owned a family company, and 10.5% only had high school education, 47.5% had a bachelor’s degree or equivalent, and 42.1% had a post-graduate degree (e.g., MBA). Our entrepreneurial sample represented several work sectors, with financial services, planning, and communications the most numerous areas (6.3% each), followed by services, logistics, administration, and biochemical (4.7% each). 7.8% of the participants did not indicate their sector. Managers mostly supervised clerical employees.

### Procedure

As participants entered the lab, the experimenter randomly assigned each participant to a cubicle. All the participants answered a self-report survey for 25 min before the laboratory task started (capturing age, anatomical sex, and entrepreneurial intentions). Immediately after, participants provided self-reports of authentic leadership and social desirability (as a consistency check).

The laboratory task consisted of several activities. First, a couple of activities collected information on our behavioral control variables. More precisely, an arithmetic exercise was used as a proxy variable of participants’ cognitive ability. A risk aversion game followed our arithmetic exercise. We conducted the risk aversion game because meta-analyses revealed that individuals displaying behaviors aligned with both the female gender and managerial role stereotype declare a higher risk aversion than those individuals displaying behaviors aligned with the male gender role and the entrepreneur role stereotype (Rauch and Frese, 2007; Stewart and Roth, 2007).

In comparison, a lower risk aversion aligns better with the female gender role and the manager role stereotypes. Finally, participants undertook our two antisocial economic behavior games (“Joy-of-Destruction” and “Cheating”). After the study, all participants were debriefed about the nature of the study and received a $5 show-up fee and their respective earnings from the economic games that comprised this study’s laboratory task. Participants’ earning ranged from $2.97 to $11.75 (*M* = $7.59; *SD* = 1.85).

For Phase 2, we employed the same procedure as in Phase 1, with a slight modification. After the risk aversion activity, we added a “one-shot” public goods game that captured participants’ preference for pro-social vs. pro-individual strategizing. A pro-social strategizing aligns with the female gender role and pro-individual strategizing with the male gender role.

Further, we felt it unnecessary to assess actual leaders and entrepreneurs’ cognitive ability. Instead, we collected an array of demographic characteristics. More precisely, we measured (a) Span of Control, meaning the number of employees supervised, (b) leading in a family company (dummy coded as 0 = “No”/1 = “Yes”), and (c) work tenure as leader, measured in years.

All participants provided informed consent to participate in the laboratory study and had no prior knowledge of the study’s objectives. Every participant was initially endowed with the same quantity of resources (100 tokens, equal to 10 EUR), allocated to a private account, and paid off at the end of the laboratory study. Total earnings were calculated as the sum of all the earnings obtained in all the games.

Participants’ earning ranged from $2.00 to $9.86 (*M* = $6.28; *SD* = 1.78). At the end of the session, we offer the possibility of exchanging their monetary payoffs with souvenirs from the university, such as coffee mugs, t-shirts, caps, and pens. Most participants preferred the souvenirs to the monetary compensation.

### Measures

#### Phase 1

##### Entrepreneurial intention

Given that our sample consisted of business students (and not actual entrepreneurs), we measured participants’ entrepreneurial intention by asking participants about the likelihood of starting a venture after graduation. The item “How likely is that you would start a venture after you graduate?” was rated on a 5-point Likert-type scale, with values ranging from “1 = “Extremely unlikely” to “5 = “Extremely likely.” Although a self-report scale of entrepreneurial intention exists (Liñán and Chen, 2009), the items that comprise the subscale of interest revealed that all items referred to the same notion. Therefore, we used a single item from Liñán and Chen’s (2009) sub-scale in this laboratory study.

##### Authentic leadership

We asked participants to self-report the frequency of their AL behaviors using the Authentic Leadership Questionnaire (Walumbwa et al., 2008). All sixteen items were rated on 5-point Likert-type scales, with values ranging from “1 = Not at all” to “5 = Frequently, if not always.” Some examples of items are “Seeks feedback to improve interactions with others” (Self-awareness), “Says exactly what he or she means” (Relational Transparency), “Makes decisions based on his/her core beliefs” (internalized moral perspective), and “Listens carefully to different points of view before coming to conclusions” (Balanced Processing of Information). Cronbach’s was α = 0.70 for the overall scale for Phase 1, and Cronbach’s α was 0.81 for Phase 2.

##### Antisocial economic behaviors

We captured two antisocial economic behaviors (harming others and cheating) by employing simplified versions of the “Joy of Destruction” (Abbink and Sadrieh, 2009) and “Cheating” games (Fischbacher and Föllmi-Heusi, 2013). Such games present players with a simplified version of real-stakes business decisions. Both games were scored with a binary outcome (i.e., 0 = “No,” 1 = “Yes”).

In the “Cheating” game (Fischbacher and Föllmi-Heusi, 2013), a player is provided with specific information about a product, and its asked to report such information to others (e.g., shareholders) for a pre-determined payoff (Dummy coded as “0”). However, when reporting said information, the player is provided with a choice, which mainly consists of misrepresenting the information provided in exchange for a higher individual payoff (dummy coded as “1”). Selecting “1” would capture a cheating behavior.

In our game, we reduced the complexity of the decision-making process by proposing a simplified version of the cheating game. Individuals were asked to report the color of a ball from an urn, knowing that the red ball reported 4 US dollars, the blue ball reported 2 US dollars, and the green ball reported no gains. Because all the balls were green in color, we measured a cheating behavior when individuals chose red and blue balls by simply asking the question of what color is the ball.

In our variation of the “Joy-of-Destruction” “game,” each player is presented with the chance to harm the competition (i.e., a player randomly matched at the beginning of the game) at no additional cost to the player’s firm (choosing “1 = Yes” captures a destruction preference). Such a decision has been validated as a measure of destructive behavior (see Abbink and Herrmann, 2011). In particular, we used a simplified version of the game in which individuals (Players A) were endowed with 3 US dollars and matched with an anonymous passive participant (Player B) which received 10 US dollars. The only question that Players A received was about to reduce the other’s endowment in 7 US dollars. All the participants played simultaneously as Player A and B for payment effects.

#### Phase 2

##### Authentic leadership

Again, participants self-reported their authentic leadership using the ALQ (Walumbwa et al., 2008; Avolio et al., 2018). Items were rated on a 5-point Likert-type scale (1 = “Not at all” to 5 = “Frequently, if not always”). Although the ALQ has been validated for the Iberian, Spanish-speaking population (Moriano et al., 2011), linguistic differences exist between Iberian and the language spoken in Costa Rica, Latin-American Spanish. Consequently, we followed Brislin (1980) guidelines to translate the original questionnaire into Latin-American Spanish. The first author, a native Latin-American Spanish speaker, translated the original items of the ALQ scale into Latin American Spanish and required a consistency check from four Latin-American research assistants (blind to the laboratory study). Finally, the translated copy was provided to an English professional translator for re-translation into English. No linguistic differences between the original and back-translated scale emerged. In Phase 2, we used the same two games employed in Phase 1 with the same decision options and pay-out functions.

### Control Variables

#### Phase 1

##### Cognitive ability

Arithmetic ability was taken as a proxy for cognitive ability (Hyde et al., 1990). Cognitive ability is a trait of successful entrepreneurs (Frese and Gielnik, 2014) and is regarded as the main predictor of performance (Schmidt and Hunter, 2003; Kanfer and Kantrowitz, 2005). Participants were asked to complete 30 simple arithmetic calculations in 30 s and were rewarded with $ 0.10 for every correct answer.

##### Social desirability

We used a 12 item scale of social desirability by Caprara et al. (1993). This construct captures a person’s tendency to display an enhanced image of him or herself. Cronbach’s Alpha was α = 0.82 for Phase 1. In Phase 2, Cronbach’s Alpha was of α = 0.85.

##### Risk aversion

Despite the findings of Filippin and Crosetto (2016), which concluded that effect of gender differences on risk aversion appears in less than 10% of their review studies, taking risks is an expected stereotypical behavior of entrepreneurs. For entrepreneurs, a higher risk aversion would likely lead to social backlash or punishment. Therefore, embracing the entrepreneur role stereotype in extremis should lead to excessive risk taking.

In our game, participants were asked to choose one of three lotteries, each with a different degree of risk which was established by throwing a virtual coin. Participants were endowed with one US dollar and were asked about not playing any lottery (option A = $1), increasing payoffs and losses by 50% (option B = $0.5/$1.5), or increasing them by 100% (option C = $0/$2). After the lottery choice (among the three options), random plays determined participant’s payoffs. The random nature of the lottery captures participants’ inability to calculate the risk of their choice. We consider a participant to be risk adverse when he or she selected option A.

#### Phase 2

For phase 2, we controlled for the participants’ demographic characteristics that might influence their economic behavior, as suggested by existing studies. Again. we controlled for (a) social desirability, (b) whether if the entrepreneur’s venture was a family business or not (coded “0” for managers as well), (c) their span of control (number of supervised employees), and (d) work tenure as leaders (for entrepreneurs and managers).

##### Pro-individual vs. pro-social strategizing

As mentioned above, we used a Public Goods Game (PGG) to capture entrepreneurs’ economic behavior that might evidence a preference for social entrepreneurship. One type of PGG is the Voluntary Contribution Mechanism (Keser, 2002; Chaudhuri, 2011). In such a cold-strategy public good game, participants create wealth by adopting pro-individual or pro-social contribution strategies.

The pro-individual strategy consists of capturing part of the shared pool without contributing substantially to the public good (and thus conforming to the entrepreneurial role stereotype). The pro-social strategy involves contributing substantially to the public good and trusting that others will contribute as well. A pro-social strategizing would evidence a preference for social entrepreneurship, as reflected by a higher contribution to the public good, that those with a pro-individual strategizing (a preference for traditional entrepreneurship). This measure ranged from “0” = pro-individuals strategizing up to “100” = pro-social strategizing.

### Data Analysis

#### Phase 1

We tested our hypotheses by building a structural equation model in MPLUS 8.0. MPLUS 8.0 allows employing robust estimators, such as the Weighted Least Squares – Mean and Variance Adjusted (WSLMV). The WLSMV allows analyzing models comprising dichotomic variables, calculates well parameters estimates with relative small datasets, and adjusts for deviations of multivariate normality (Moshagen and Musch, 2014).

To assess our SEM model’s fit, we employed the Satorra-Bentler scaled chi-square test and additional Goodness of Fit Indicators. The S-B chi-square test indicates a good model fit when it is non-significant (Geiser, 2011), with the caveat that the S-B chi-square test is sensitive to large sample sizes. Therefore, it is a good practice to complement the S-B chi-square test with additional goodness-of-fit indicators. Some examples are the χ2/df ratio, the Root Mean Square Error of Approximation (RMSEA), the Comparative Fit Index (CFI), and the Tucker-Lewis indicator (TLI), as well as and the Standardized-Root-Mean-Square-Residual (SRMR).

The comparative fit index (CFI) measures incremental fit whereby values higher than 0.90 and ideally above 0.95 are required to avoid incorrectly accepting miss-specified models. Similarly, the TLI is an indicator of model parsimony equivalent to the NNFI. Again, values above 0.90 (and ideally above 0.95) are preferred. CFI and TLI values close to 1 indicate that the model explains the data better than an independence model (Hu and Bentler, 1999). The RMSEA tests for approximate data fit, and it should be at least equal to 0.08 or below. Standardized-Root-Mean-Square-Residual (SRMR) provides an overall evaluation of the residuals and it is considered acceptable when it approximates the 0.08 value (Hu and Bentler, 1999).

Finally, we parceled any multi-dimensional measure in our study. Parceling refers to aggregating the respective items of a scale’s dimension to reduce the overall parameters to be estimated in an SEM (see Monzani et al., 2021a; Seijts et al., 2021 for examples of parceling), and thus. Whereas this technique has received some critiques, Little et al. (2009) argued that parceling is justified when the underlying factorial structure has been previously established in the literature and when the parceled indicators respect said factorial structure. Given that both our Social desirability measure and the ALQ have been validated in multiple samples worldwide (Walumbwa et al., 2008), their parceling is justified.

#### Phase 2

We used SPSS 25 to conduct hierarchical binary logistic regressions. To avoid multicollinearity, we normalized scores for all our continuous independent variables before computing any interaction term. Anatomical sex was coded into 0 = “Male” and 1 = “Female,” and entrepreneurial status as 0 = “Manager” and 1 = “Entrepreneur.”

We mainly entered our demographic and control variables (social desirability; Family Company; Span of control; and Work tenure; pro-social strategizing; Risk Aversion and either Cheating or Joy-of-Destruction, respectively). Then, we entered our predictors (entrepreneurial intentions, biological gender, and authentic leadership scores). Finally, we included our two cross-product terms. We used Dawson (2013) Microsoft Excel templated to illustrate any non-linear interaction effects.

Given that a binary logistic regression uses a maximum likelihood approach, SPSS 25 provides goodness-of-fit indices. These indices allow assessing if (a) a model correctly classifies predicted cases into their observed categories, (b) how well the model fits the observed data, (c) and whether if each model step improves the fit of the model to the observed data in hierarchical models. First, the cutoff value to evaluate the sensitivity of a model is 50%. Higher percentage scores represent a higher sensitivity of the model. In social sciences, a test sensitivity of 50–60% is considered poor, from 60 to 70% is adequate, 70 to 80% is good, and above 80% is very good. Any percentage equal to or below 50% would mean that the model has equal or fewer chances of classifying cases correctly than a coin toss.

The second goodness of fit indicator is the omnibus test of the model. The omnibus test captures how much our model deviates from a null model (a model only with the intercept and no additional variables) or the previous step if a hierarchical regression approach is used. For this indicator, higher χ^2^ scores indicate a better fit; a statistically significant χ^2^ value would indicate that such deviation did not occur by chance.

The third set of goodness of fit indicators is based on the deviance statistic (–2LL), which follows a chi-square distribution. Researchers employ –2LL statistic to derive pseudo-R^2^ statistics (e.g., Cox & Snell R^2^ and Nagelkerke R^2^). In short, these two statistics range from 0 to 1 and indicate in relative terms how well a model fits the data, which scores closer to 1 suggesting a better fit. Fourth, the Hosmer & Lemeshow test is analog to the χ^2^ test used in SEM modeling. The *lower* that χ^2^ score is, the better that the model fits the observed data. Finally, a non-significant p-value would indicate that the model fits the data well.

## Results

### Phase 1 – Hypothesis Testing

**FIGURE 2:**
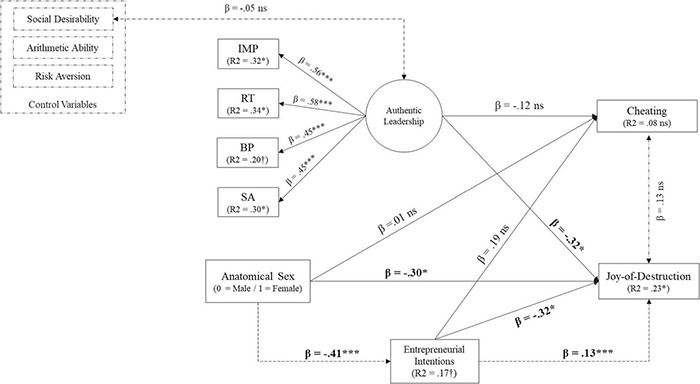
Phase 1 – Revised main and indirect effects model. ***p < 0.0001; *p < 0.05; ^†^*p* < 0.10; ns = non-significant.

Table 1 shows Means, Standard Deviations, and both Pearson’s *r* (product-moment correlation) and Kendall’s τ (tau) in the upper and lower diagonals, respectively. Entrepreneurial intentions were negatively related to the female anatomical sex (Kendall’s τ = –0.22^∗^). Figure 2 shows the standardized coefficients resulting from our SEM analysis. For parsimony, only significant paths are shown. Further, whereas solid lines represent main effects, dotted lines represent either indirect effects or corrected (or latent) correlations between our constructs.

**TABLE 1:** Phase 1 – Means, standard deviations, Kendall’s τ and Pearson’s r for all study variables.

		***M***	***SD***	**0.1**	**0.2**	**0.3**	**0.4**	**0.5**	**0.6**	**0.7**
(1)	Cognitive Ability	1.15	0.38	–	–0.18	0.20	–0.01	0.11	0.01	0.11
(2)	Risk Aversion	1.49	0.50	–0.14	–	–0.13	–0.03	–0.11	0.12	0.07
(3)	Anatomical Sex	0.74	0.44	0.10	–0.13	–	–0.20	0.06	–0.13	–0.04
(4)	Entrepreneurial Intention	3.45	1.08	0.03	0.01	−0.22*	–	0.10	–0.15	0.14
(5)	Authentic Leadership	2.96	0.33	0.10	–0.10	0.06	0.05	–	–0.20	–0.07
(6)	Antisocial Behavior-Joy of Destruction game	0.61	0.49	0.04	0.12	–0.13	–0.16	–0.18	–	0.14
(7)	Antisocial Behavior-Cheating game	0.59	0.50	0.12	0.07	–0.04	0.12	–0.07	–0.14	–

*^∗^*p* < 0.05. The lower diagonal presents parametric correlations in the upper diagonal (Pearson’s r) and non-parametric correlations in the lower diagonal (Kendall’s tau) given that several variables are dichotomous.*

The results of our SEM analysis revealed that in overall, our model fit showed an excellent fit to the data (χ^2^_(33)_ = 38.87, ns; χ^2^/df = 0.93; RMSEA = 0.0001; CFI = 1.00; TLI = 1.00; SRMR = 0.11). In consequence, the standardized effect sizes and standard errors derived from this model are trustworthy.

A detailed inspection of Figure 2 shows that after controlling for the effects of all the other variables in our model, our two dependent variables (participants’ choices in the “Joy-of-Destruction” and Cheating games) were not correlated [*r* = 0.14 (0.19), *ns*]. Further, predictors majorly explained a statistically significant amount of variance for the “Joy-of-Destruction” game (R^2^ = 0.23, *p* < 0.05), but not for the cheating game (R^2^ = 0.23, *p* < 0.05).

Second, neither of our control variables (Social desirability; Arithmetic ability, nor a Risk Aversion preference) had statistically significant main effects on neither the “Joy-of-Destruction” nor the Cheating games. Instead, whereas none of our independent variables were significant predictors of participants’ behavioral choices in the cheating behavior, all three independent variables were significant (and negative) predictors of participants’ behavioral choices in the “Joy-of-Destruction” game. More precisely, Anatomical Sex [β = –0.30 (0.15), *p* < 0.05]; Entrepreneurial Intentions [β = –0.32 (0.12), *p* < 0.01] and Authentic leadership [β = –0.32 (0.15), *p* < 0.05] reduced the likelihood of observing an antisocial behavior aimed at harming one’s competition. When taken as a whole, these results support Hypotheses 1a, 2a, and 3a but do not support Hypothesis 1b, 2b, and 3b.

### Phase 1 – *Post hoc* Analyses

We conducted additional two *post hoc* analyses as per the results of our SEM model. First, we attempted to replicate the prior findings in the entrepreneurship literature regarding gender differences in entrepreneurial intentions (Jennings and Brush, 2013). To this end, we specified an additional (non-hypothesized) path between participants’ Anatomical sex and their self-reported Entrepreneurial Intentions.

We expected to find a negative effect of Anatomical Sex on Entrepreneurial Intentions because female participants were dummy coded into the “1” category. Our results revealed that Anatomical Sex had a relatively strong negative effect on Entrepreneurial Intentions (β = –0.94; *p* < 0.01). This result means that in our sample, female participants tended to declare a weaker intention of starting up a business after they graduate from their business programs. Second, we attempted to integrate this non-hypothesized finding with our prior results. To this end, we tested if Anatomical Sex would have an indirect effect on participants’ behaviors choices for the “Joy-of-Destruction” game (recall that we did not find a main effect of Anatomical Sex on behavioral choices on the Cheating game). By using the INDIRECT function in MPLUS 8.0, we detected a significant and positive indirect effect of Anatomical Sex on participants’ behavioral choices on the “Joy-of-Destruction” game, as mediated by Entrepreneurial Intentions (β = 0.13 (0.06); *p* < 0.05). In other words, those female participants who see themselves as entrepreneurs in the future seem to embrace the ultra-competitive prescriptions of the entrepreneurial role stereotype.

Our findings of main and indirect effects with opposing signs align with our theorizing. This last result evidences the psychological tension that women declaring entrepreneurial intentions suffer. Women chose not to hurt their competition in the Joy-of-Destruction game (as prescribed by the female gender role). However, this choice was nuanced by a weaker yet positive and significant indirect effect (mediated by entrepreneurial intentions), and likely driven by participants’ stereotypical views of entrepreneurship.

### Phase 2 – Hypothesis Testing

Table 2 shows the means, standard deviations, Pearson’s and Kendall’s correlations for all variables in this study. Being an entrepreneur was strongly and positively correlated with having a family company (*τ* = 0.75^∗∗^) and the time leading others (*τ* = 0.41^∗∗^), but negatively correlated with Span of Control (*τ* = –0.27^∗^) and self-ratings of authentic leadership (*τ* = –0.27^∗∗^).

**TABLE 2:** Phase 2 – Means, standard deviations, Kendall’s tau and Pearson’s bivariate correlations for all study variables.

		***M***	***SD***	**1**	**2**	**3**	**4**	**5**	**6**	**7**	**8**	**9**	**10**
(1)	Family Company	0.20	0.40	–	−0.32*	0.50**	–0.08	–0.03	–0.01	0.75**	−0.35**	–0.17	–0.16
(2)	Span of Control	15.86	17.72	−0.35*	–	–0.22	–0.04	0.05	–0.12	−0.26*	0.12	–0.16	0.02
(3)	Work Tenure (as Leader)	7.55	7.68	0.30**	–0.14	–	–0.10	–0.08	–0.08	0.51**	–0.05	−0.27*	–0.20
(4)	Risk Aversion	1.32	0.47	–0.08	–0.05	–0.10	–	–0.16	–0.16	–0.08	0.12	–0.08	0.15
(5)	Social Desirability	0.94	0.45	–0.16	0.01	–0.13	–0.16	–	0.10	–0.12	−0.25*	0.01	–0.14
(6)	Anatomical Sex	0.47	0.50	–0.01	–0.03	–0.02	–0.16	–0.03	–	0.11	–0.15	–0.05	–0.24
(7)	Entrepreneurial Status	0.31	0.47	0.75**	−0.27*	0.41**	–0.08	–0.12	0.11	–	–0.33	–0.19	–0.17
(8)	Authentic Leadership	3.31	0.34	−0.28**	0.14	0.01	0.09	0.08	–0.10	−0.27*	–	–0.12	–0.03
(9)	Antisocial Behavior-Joy of Destruction game	0.39	0.49	–0.16	–0.04	−0.22*	–0.07	–0.05	–0.05	–0.19	–0.11	–	0.07
(10)	Antisocial Behavior-Cheating game	0.20	0.41	–0.16	–0.14	−0.25*	0.15	0.07	–0.24	–0.17	–0.05	0.07	–

***p < 0.0001; **p < 0.01; *p < 0.05; The lower diagonal presents parametric correlations in the upper diagonal (Pearson’s r) and non-parametric correlations in the lower diagonal (Kendall’s tau) given that several variables are dichotomous in nature.

The left panel of Table 3 shows the results of our logistic regression model predicting participants’ likelihood of choosing to harm others’ firms. Our model was trustworthy and had good sensitivity (73.8%), significantly deviated from the null model (χ^2^_(__12__)_ = 25.79^∗^) and fitted the observed data well (H&L Test = χ^2^_(__8__)_ = 8.14 ns). Three control variables were significant predictors. More precisely, Social Desirability [*B* = –0.93, (0.47); Wald’s *Z* = 3.98^∗^], owning a family company [*B* = –5.40, (2.80); Wald’s *Z* = 3.72^∗^] and work tenure [*B* = –1.78, (0.88); Wald’s *Z* = 4.14^∗^] reduced the likelihood of choosing to harm others’ firms (“Joy-of-Destruction”).

**TABLE 3:** Phase 2 – Logistic Regression model predicting the likelihood of displaying two antisocial behaviors (*N* = 62).

	**Joy-of-Destruction Game**					**Cheating Game**				
	**β**	**SE β**	**Wald’s Z**	**df**	**Exp (β)**	**β**	**SE β**	**Wald’s χ^2^**	**df**	**Exp (β)**
Constant	3.15	1.35	5.47*	1	23.26	–0.52	0.86	0.37	1	0.36
Social Desirability	–0.93	0.47	3.98*	1	0.39	–0.08	0.49	0.30	1	0.92
Family Company	–5.40	2.80	3.72*	1	0.01	–2.09	2.79	0.56	1	0.12
Span of Control (N Employees)	–1.09	0.58	3.58†	1	0.34	–0.14	0.38	0.13	1	0.87
Work Tenure	–1.78	0.88	4.14*	1	0.17	–1.32	0.87	2.30	1	0.27
“Cheating” Behavior (1 = Yes)	–1.04	0.92	1.28	1	0.35	–	–	–	–	–
“Joy-of-Destruction” Behavior (1 = Yes)	−	–	–	–	–	–0.60	0.90	0.46	1	0.54
Risk Aversion (1 = High)	–2.01	0.89	5.06*	1	0.13	–0.09	1.02	0.01	1	0.91
CPG	–0.52	0.37	2.00	1	0.59	–0.80	0.47	2.81†	1	0.12
ES – (1 = Entrepreneur)	0.93	1.33	0.49	1	2.53	0.53	1.56	0.11	1	1.69
AS (1 = Female)	–1.81	0.85	4.56*	1	0.16	–2.14	0.98	4.77*	1	0.73
Authentic Leadership	–1.68	0.80	4.41*	1	0.19	–1.90	0.95	3.97*	1	0.15
Authentic Leadership x BG.	2.44	1.05	5.35*	1	11.49	2.76	1.31	4.43*	1	15.73
Authentic Leadership x ES.	–2.99	1.51	3.89*	1	0.05	–1.01	1.77	0.33	1	0.36
Goodness-of-fit Indicators										
Correctly Classified Cases			73.8%							77.0%
Deviation from Null Model			χ^2^_(12)_ = 25.79**							χ^2^_(12)_ = 25.79**
Hosmer & Lemeshow Test			χ^2^_(8)_ = 8.14 ns							χ^2^_(8)_ = 11.08 ns
Pseudo R^2^	–2LL =	56.78	C&S R^2^ = 0.34		N – R^2^ = 0.46	–2LL = 56.78		-2LL = 45.06		C&S R^2^ = 0.26

†*p* < 0.10; *p < 0.05; **p < 0.01; All continuous variables were standardized. AS, Anatomical Sex; ES, Entrepreneurial Status; CPG, Contribution to the Public Good; C&S – R^2^, Cox and Snell pseudo R^2^; N – R^2^, Nagelkerke’s pseudo R^2^. The accepted cut-off for case classification is 50%. Scores above 70% evidence a good classification ability of the model. Similarly, A non-significant score in the Hostmer & Lemeshow test suggest a good fit of the model to the data.

**FIGURE 3:**
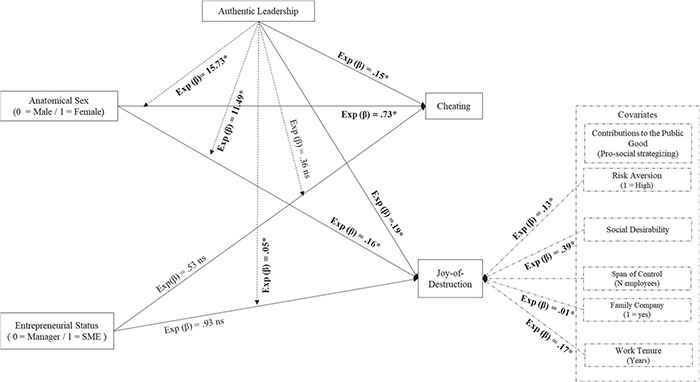
Phase 2 – Main and interactive effects model (Logistic Regression). *p < 0.05; ns = non significant.

Regarding the main effects of our independent variables, Anatomical Sex [*B* = –1.81, (0.85); Wald’s *Z* = 4.56^∗^] was again a negative predictor of a “Joy-of-Destruction” preference, suggesting that men are more likely to choose to harm others’ firms than women. Instead, our participants’ Entrepreneurial status did not have a main effect [*B* = 0.93, (1.33); Wald’s *Z* = 0.49 ns]. Finally, like Anatomical Sex, Authentic leadership had a negative effect [*B* = –1.68, (0.80); Wald’s Z = 4.41^∗^], meaning as the frequency of authentic leadership behaviors decreased, the more likely participants were to choose to harm others’ firms. Overall, these results support H1a and H3a but again do not support H2a (see Figure 3).

**FIGURE 4:**
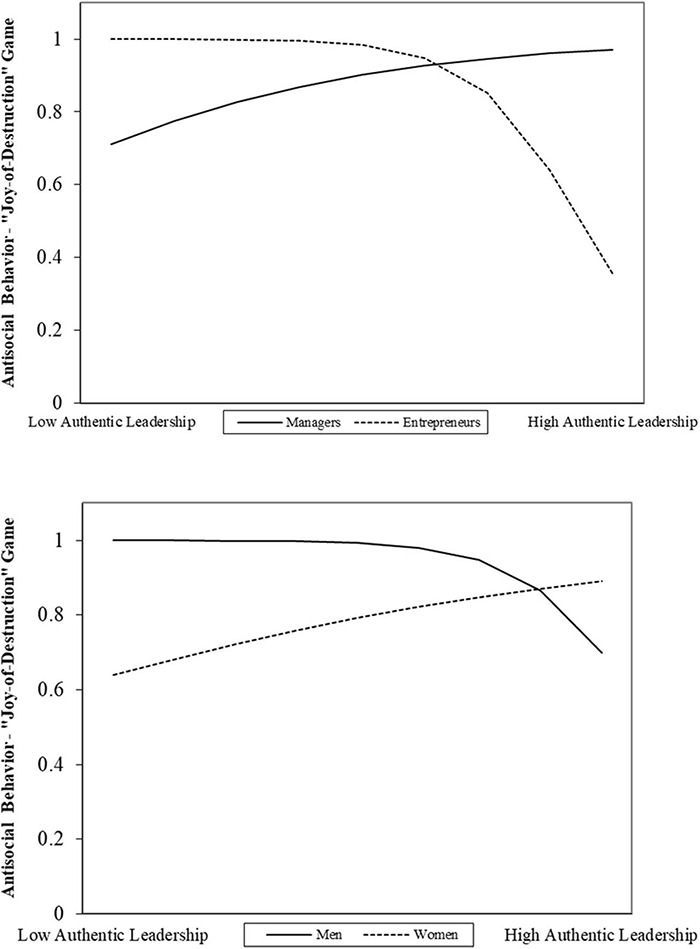
Interactive effects of authentic leadership and entrepreneurial status (Upper) and anatomical sex (Lower) on the likelihood of an affirmative decision in the “Joy-of-Destruction” game.

Both interaction terms predicting the “Joy-of-Destruction” preference were statistically significant. Anatomical Sex [*B* = 2.44, (1.05); Wald’s Z = 5.35^∗^] and Entrepreneurial status [*B* = –2.99, (1.51); Wald’s *Z* = 3.89^∗^] interacted with Authentic leadership in reducing participants’ likelihood of choosing to harm others’ firms. More precisely, as either men or entrepreneurs scored higher in authentic leadership, the likelihood of harming others’ firms decreased. Figure 4 illustrates these moderator effects. These results provide initial support for hypothesis 4a and 5a.

The right panel of Table 3 shows the results of our second logistic regression predicting the likelihood of participants’ cheating. Our model showed good sensitivity (77.0%), and this time, it significantly deviated from the null model (χ^2^_(__12__)_ = 25.79) and fitted the observed data well (H&L Test = χ^2^_(__8__)_ = 11.08 ns). None of our control variables were significant predictors. Instead of our independent variables, again Anatomical Sex [*B* = –2.14, (0.98); Wald’s *Z* = 4.77^∗^] was a negative predictor, suggesting that, in general, men are more likely to cheat than women. Something similar occurred for Authentic leadership [*B* = –1.90, (0.95); Wald’s *Z* = 3.97^∗^], meaning that as the frequency of authentic leadership behaviors increased, participants were less likely to cheat. Finally, our participants’ entrepreneurial status did not have a main effect [*B* = 0.53, (1.56); Wald’s *Z* = 0.11 ns]. Thus, our results support H1b but do not support H2b.

**FIGURE 5:**
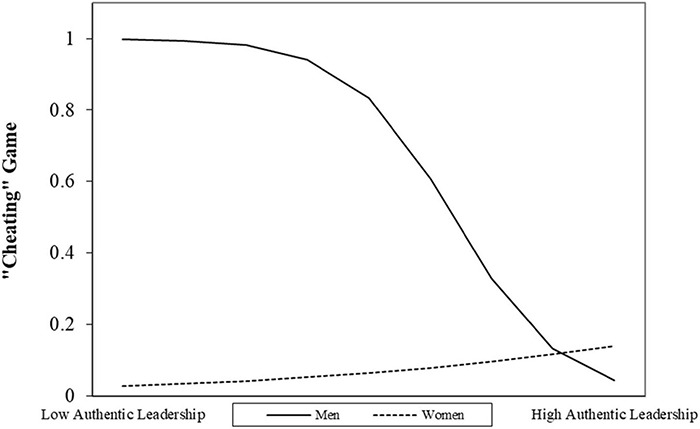
Interactive effects of Authentic Leadership and Anatomical Sex on the likelihood of an affirmative decision in the “Cheating” Game.

Figure 5 shows that only Anatomical Sex [*B* = 2.76, (1.31); Wald’s *Z* = 4.43^∗^] interacted with Authentic leadership in reducing the likelihood of participants’ cheating. As men scored higher in Authentic leadership, the likelihood of participants cheating decayed. Thus, we found support for H4b but not for H5b.

## Discussion

Our study’s main goal was to explore whether if role stereotypes drive entrepreneurs to engage in antisocial economic behaviors. More precisely, we proposed extending Eagly and Karau’s (2002) RCT to the entrepreneurial arena and testing the existence of a potential entrepreneurial role stereotype, as well as a female-entrepreneurship role conflict (Laguía et al., 2018). The results of Phase 1 revealed that female business school students tend to declare weaker entrepreneurial intentions than men. Further, women are less likely to choose to harm their competitors in an economic game (“Joy-of-Destruction”). However, a *post hoc* analysis revealed that this reluctance to harm other firms is reduced when entrepreneurial intentions mediate this link.

Building on Eagly and Karau’s (2002) theory, we claim that the entrepreneur role stereotype captures dominant traits and prescribes competitive behaviors that align with the male gender role stereotype. Still, we distinguish it from the assertive traits and behaviors prescribed by the leader role stereotype. Consequently, we propose two new role incongruences, namely, the gender-entrepreneur and leader-entrepreneur incongruencies.

In short, the female-entrepreneur incongruence would explain why women (or those persons that identity with the female gender role) resist occupying entrepreneurial roles (Jennings and Brush, 2013), a result we confirmed in Phase 1. Further, the female-entrepreneur incongruence would explain why those women that occupy an entrepreneurial role tend to gravitate toward communal-oriented ventures instead of pursuing ventures in more traditional sectors (Datta and Gailey, 2012).

Instead, although not tested in this work, we argue for a leader-entrepreneur role conflict that would explain why some individuals become serial entrepreneurs (rejecting the leader role prescriptions of managing a venture to its mature state), and other entrepreneurs eventually gravitate into managerial roles in other’s firms (rejecting the entrepreneur role prescription of creating wealth through a new venture creation).

A core premise of this study is that self-stereotyping and embracing a role stereotype “in extremis” is a dysfunctional way of reducing these role incongruencies. Our results suggest that women or entrepreneurs who do so will likely end up displaying antisocial economic behaviors characteristic of a “toxic masculinity” mindset, given the overlap between the male gender role and both the leader and entrepreneur roles. The second premise of our work was that displaying positive leadership behaviors (regardless of one’s hierarchical position) might be a better alternative to reduce psychological tension than embracing a stereotypical role in-extremis. The results of Phase 2 show that for entrepreneurs and males, high scores in authentic leadership reduced the effects of role stereotypes on antisocial behaviors.

Digging deeper into our findings, our model predicted that the more strongly than participants embraced the agentic behaviors of the male role stereotype (H1a, H1b) or the hyper-competitive prescriptions of the entrepreneur role stereotype (H2a, H2b), said participants would be more likely to prefer (and display) antisocial behaviors, such as cheating or harming others’ firms to get ahead. We tested such a claim using two realistic economic games (The “Joy-of-Destruction” and the “cheating” game) that would evidence said toxic masculinity mindset.

The results of Phase 1 provide mixed support for our predictions. In line with RCT, women are less likely to display entrepreneurial intentions and engage in antisocial economic behaviors to harm their competition (H1a). This finding aligns with the prior literature on female entrepreneurship (Gupta et al., 2009; Laguía et al., 2018). Similarly, as predicted by our main effects model, as the self-reported frequency of authentic leadership behaviors increases, participants’ likelihood of choosing to harm others’ firms decreases (H3b). Again, this result aligns with reports in the positive leadership literature, which related authentic leadership to ethical and pro-social behaviors in work contexts (Hannah et al., 2011, 2014).

Our results show two counter-intuitive findings. The first counter-intuitive finding was that the more willing participants were to start up a firm, the less likely they were to harm others’ firms (H2a). This behavior deviates from the prescription for the entrepreneur role stereotype. We invoke a sample effect as an alternative explanation for this finding. In other words, declaring entrepreneurial intentions does not equate to occupying an entrepreneurial role. Thus, participants might have made decisions in our economic games based on their implicit stereotypical role expectations about how entrepreneurs should behave without experiencing the psychological tension that results from simultaneously occupying an entrepreneur and leader role.

The second counter-intuitive finding is that we anticipated that the more that participants would see themselves as authentic leaders, the less likely they would be to cheat, but that did not occur. A possible explanation might come from the Sendjaya et al. (2014) study, showing that as their participants’ Machiavellism scores increased, the link between authentic leadership and moral action was reversed.

Again, an alternative explanation for these last findings might exist. Participants of Phase 1 comprised a heterogeneous sample of business school students and thus not “real-life” leaders. Such participants were socialized in cultures with opposing values regarding the social expectations for gender, leader, and entrepreneur role stereotypes. Thus, we decided to re-test our model in a more homogeneous sample, which ideally would comprise real entrepreneurs and managers, and conduct such a study in a western culture that embraces more traditional values than France.

Seeking to test potential mitigations for these role conflicts, in Phase 2, we adopted a gendered view of leadership. More precisely, we claimed that any given androgynous leadership style would reside at the center of the agency-communal continuum proposed by RCT. Therefore, said androgynous leadership behaviors would mitigate the toxic effect that the toxic masculinity inherent to the male gender role stereotype has on antisocial economic behaviors without triggering the double bind explained by RCT.

Following the above logic and extant research, we predicted that adopting an authentic leadership style would negatively relate to antisocial behaviors (H3a and H3b). Further, we predicted that adopting an authentic leadership style would enable men (H4a, H4b) and entrepreneurs (H5a, H5b) to deviate enough from the stereotypical mandates of their role stereotypes without fear of a social backlash, reducing the likelihood of observing antisocial behaviors in our games. In other words, we expected Authentic leadership to reduce the female-leader and the leader-entrepreneur role conflicts, respectively. All our hypotheses involving authentic leadership were confirmed, except one (Hypothesis 3b).

In short, our results revealed that only the effect of being anatomically male sex on the “Joy-of-Destruction” game was significant across studies. This finding evidences the negative effect of embracing stereotypical male behaviors for aspiring or actual entrepreneurs, given the inherently unethical and unsustainable nature of these practices.

### Implications for Theory

Our work provides a valuable theoretical contribution to the field of leadership and the domain of female entrepreneurship. First, our work answers the call of moving beyond the study of anatomical differences in gender-focused entrepreneurship research and avoiding other forms of invisible prejudice, as purely associating female entrepreneurs with gender-congruent ventures, also unfortunately known as the “pink ghetto” (Jennings and Brush, 2013; Carter et al., 2015). Thus, the first contribution of our work is extending the RCT into the entrepreneurship arena. We contribute to RCT by proposing two additional role incongruencies, the leader-entrepreneur, and the female-entrepreneur role incongruencies.

We claim that a triple bind and prejudice derives from unpacking the male role stereotype characteristics, mainly agentic and competitive traits (Mollaret and Miraucourt, 2016). We claim that the agentic traits of the male gender role would then overlap with a leader role stereotype. Instead, the male gender competitive characteristics would overlap with the entrepreneurial role stereotype. Our model has the potential of helping male and female entrepreneurs in either traditional or social entrepreneurial roles. More precisely, we believe that our insights might help entrepreneurs resist the implicit social pressures pushing toward displaying antisocial economic behaviors.

Our theorizing is novel in claiming that the mechanism that operates against women when occupying leadership positions might also apply to entrepreneurs in general. However, this mechanism acts more strongly for female entrepreneurs. For example, in addition to being expected to be visionary and managerial at the same time (leader-entrepreneur incongruence); female entrepreneurs are also expected to be assertive and caring at these same time (gender-leader role congruence), as well as self-oriented and hyper-competitive as entrepreneurs but group-oriented and cooperative as women (gender-entrepreneur role incongruence). Such conflicting expectations can explain why women resist occupying entrepreneurial roles more accurately than focusing merely on anatomical differences.

A second theoretical contribution is that we propose an update to RCT to include the new uplifting leadership theories (Hernandez et al., 2011). Many of these uplifting theories do not fit nicely into the agency-communal continuum. We focused on authentic leadership, a new genre form of leadership that claims to be the root notion underlying positive forms of leadership for many scholars (Avolio and Gardner, 2005). Our model acknowledges and honors the RCT, at the same time proposes a more integrative gendered view of leadership, given that AL is neither fully agentic nor entirely communal (Monzani et al., 2015).

Currently, RCT focused mainly on the full-range leadership theory to describe the transactional leadership style as aligning with the agentic prescriptions of the male role stereotype and the transformational leadership style as aligning with the communal prescriptions of the female role stereotype. Thus, our work might inspire future research studies to explore how would other positive leadership styles, such as Ethical leadership (Brown et al., 2005) or Servant Leadership (Eva et al., 2019), or even Identity Leadership (Steffens et al., 2014; van Dick et al., 2018) connect with the predictions of RCT.

Finally, a third theoretical contribution of our study explains why traditional entrepreneurs tend to display antisocial economic behaviors. We focused on unfair competition or faking shareholder reports and product information as defined in microeconomic behavior studies. The importance of finding new insights on preventing the display of such antisocial economic behaviors cannot be overstated. Entrepreneurial integrity matters because whereas such antisocial economic behaviors might be functional for the short-term success of a venture, they are inherently unsustainable. So, if antisocial behaviors are institutionalized early in the life cycle of a venture, such unethical business practices will be reproduced through socialization processes as the venture matures. If unchecked, such unethical business practices will eventually erode a venture’s viability (Collewaert and Fassin, 2013).

### Implications for Practice

The first implication of our work is that it can inform policies aimed at fostering female entrepreneurship. We join Carter et al. (2015) call to move beyond just using anatomical sex as the sole criterion to promote female entrepreneurship. Further, we invite policy-makers to just stop simply “throwing money at women so that they can start a business” and adopt a broader perspective on gender identity. Although providing financial support to women and other minority groups is desirable and necessary, our results call for additional considerations.

Our results suggest that female entrepreneurship policies would be much more effective if said policies would incorporate provisions to reduce the gender-entrepreneur conflict and the leader-entrepreneur conflict. For example, besides providing funding and mentoring, policymakers could include provisions to build “entrepreneurial communities of practice” within their program participants to overcome the gender-entrepreneur role conflict. In such entrepreneurial communities of practice, female entrepreneurs could connect among themselves (or any who identify with the female gender role). Instead of harming their competitors, in such a safe space, female entrepreneurs could share knowledge, social support, and best practices without fear of a social backlash.

At a more meso-level, our work has implications for entrepreneurial strategizing. First, our work provides insights about how to prevent entrepreneurs from engaging in antisocial economic behaviors and indirectly how to prevent such behaviors from becoming embedded in their firms’ cultures as they progress through their life cycle. In other words, our work gives a valuable first step toward the primary prevention of practices that destroyed the wealth of Theranos’ shareholders.

The third practical implication is at the micro, individual level and involves the importance of positive leadership for reducing antisocial economic behavior, regardless of anatomical sex or entrepreneurial status. Creating programs to develop entrepreneurial authenticity might be useful for entrepreneurs in general and female entrepreneurs in particular. Empowering entrepreneurs to be authentic can prevent the public scandals that work against equality in entrepreneurship.

### Limitations

Like any other study, our work is not without limitations. The first limitation was that we did not manipulate any of our three exogenous variables. In other words, we presented a different type of participant (male vs. female; entrepreneurs vs. leaders) with the same economic scenario, so we cannot claim to have conducted an experimental study but a laboratory study instead. Future studies should attempt to replicate our findings by comparing participants’ behaviors against a more “hostile” economic context; for example, by adding a treatment condition that enhances or hinders the importance of individual contributions (e.g., punishment condition for antisocial behaviors).

The second limitation of our study is that we acknowledge two caveats regarding our samples. Strictly speaking, we did not have balanced samples in the laboratory studies comprising phase 1 and phase 2. However, future studies should attempt to replicate our work employing larger sample sizes balanced across conditions (Anatomical Sex, Entrepreneurial status, and so forth). However, we tried to attenuate this limitation using a robust estimator in our SEM model (WLSMV). Similarly, we employed additional goodness-of-fit indicators in our logistic regression models to ensure they were trustworthy.

The third limitation of our study is that we only focused on one developed country, namely France, and one emerging country, Costa Rica. Whereas Costa Rica could be seen as a paradigmatic case for Latin America, future studies should attempt to replicate our findings in a broader array of cultures, which might adopt and reward different cultural values.

Finally, our study only focused on one aspect of positive leadership, mainly authenticity. A more comprehensive study on what determines a positive entrepreneurial ethos besides authenticity would be essential (Hannah and Avolio, 2011; Crossan et al., 2017). A deeper understanding of what makes an entrepreneurial ethos might enlighten how developing entrepreneurial character can support entrepreneurs to display “ethics beyond expectations.”

## Data Availability Statement

The raw data supporting the conclusions of this article will be made available by the authors, without undue reservation.

## Ethics Statement

The studies involving human participants were reviewed and approved by the Instituto Tecnológico de Costa Rica, LESSAC, Burgundy School of Business. The patients/participants provided their written informed consent to participate in this study.

## Author Contributions

LM and GM conceived the presented idea and developed the experimental design. JMV organized the laboratory sessions in Costa Rica. GM organized the laboratory sessions in France and ran the sessions. LM and AH wrote the theory section. LM coordinated the statistical analysis with GM. All authors discussed the results and contributed to the final manuscript.

## Conflict of Interest

The authors declare that the research was conducted in the absence of any commercial or financial relationships that could be construed as a potential conflict of interest.

## Publisher’s Note

All claims expressed in this article are solely those of the authors and do not necessarily represent those of their affiliated organizations, or those of the publisher, the editors and the reviewers. Any product that may be evaluated in this article, or claim that may be made by its manufacturer, is not guaranteed or endorsed by the publisher.
